# DIETARY MANAGEMENT FOR DYSLIPIDEMIA IN LIVER TRANSPLANT RECIPIENTS

**DOI:** 10.1590/0102-6720201600040008

**Published:** 2016

**Authors:** Andressa S. PINTO, Marcio F. CHEDID, Léa T. GUERRA, Daiane D. CABELEIRA, Cleber D. P. KRUEL

**Affiliations:** 1 Postgraduate Program in Surgical Sciences;; 2 Division of Gastrointestinal Surgery and Liver and Pancreas Transplantation;; 3 Unit of Dietary Therapy, Hospital de Clínicas de Porto Alegre, Federal University of Rio Grande do Sul, Porto Alegre, RS, Brazil

**Keywords:** Liver transplantation, Dyslipidemia, Dietary, Diet therapy.

## Abstract

**Background:**

Dyslipidemia occurs in approximately 70% of all liver transplant (LT) recipients, and no prior control studies have demonstrated any dietary intervention to change it.

**Aim:**

To analyze the effects of a dietary intervention on the lipid profile of dyslipidemic LT recipients.

**Methods:**

All LT recipients with dyslipidemia on clinical follow-up were enrolled. Anthropometric evaluation, food history, body composition (bioimpedance) and assessment of basal metabolism through indirect calorimetry were performed. Patients met with a dietitian and an individualized diet based on estimate of basal metabolism and consisting of 25% of the total energy value in total fat and <200 mg/day of cholesterol was prescribed. Total cholesterol (TC), HDL-cholesterol (HDL), LDL-cholesterol (LDL), triglycerides (TG) and anthropometric measures were measured at baseline and six months after intervention.

**Results:**

Fifty-thee out of 56 patients concluded follow-up; age was 59±10 years; 29 were men (51.8%). The analysis pre- and post-intervention were, respectively: TC 238.9±30 and 165.1±35, p<0.001; LDL 154±33 and 90±29, p<0.001; and TG 168 (IQR=51-200) and 137 (IQR=94-177), p=<0.001. They were all modified at six months following intervention. At baseline, none of the patients had normal TC, and only 12 (22.7%) had optimal/near optimal LDL. Following dietary intervention, 45 patients (84.9%) reached normal TC and 50 (94.4%) had optimal/near optimal LDL. HDL and anthropometric measures were not modified.

**Conclusions:**

Dietary counseling with prescription of individualized diet based on estimate of basal metabolism through indirect calorimetry was able to manage dyslipidemia in most LT recipients; so, all dyslipidemic LT recipients must be enrolled on a dietary program.

## RESUMO

**Racional:**

A dislipidemia ocorre em aproximadamente 70% de todos os pacientes transplantados de fígado em acompanhamento ambulatorial. Não há relato prévio de qualquer intervenção dietética que houvesse controlado a dislipidemia nesse grupo de pacientes.

**Objetivo:**

Analisar os efeitos de uma intervenção dietética no perfil lipídico de pacientes transplantados hepáticos dislipidêmicos em acompanhamento ambulatorial.

**Métodos:**

Foram incluídos todos os pacientes adultos transplantados hepáticos com dislipidemia e em acompanhamento ambulatorial em nossa instituição. Avaliação antropométrica, anamnese alimentar, composição corporal (bioimpedância) e cálculo do metabolismo basal (calorimetria indireta) foram realizados. Pacientes foram atendidos por uma nutricionista e uma dieta individualizada baseada no metabolismo basal e consistindo de 25% do valor energético em gorduras totais e menos de 200 mg/dia de colesterol foi prescrita. Colesterol total (CT), HDL-colesterol (HDL), LDL-colesterol (LDL), triglicerídeos (TG) e medidas antropométricas foram medidos antes do início da dieta, sendo repetidos seis meses após o início da intervenção dietética.

**Resultados:**

Cinquenta e três pacientes concluíram o seguimento e tinham idade 59±10 anos e 29 eram homens (51,8%). CT pré-intervenção=238,9±30; pós-intervenção=165,1±35, p<0.001; LDL pré-intervenção=154±33; pós-intervenção=90±29, p<0.001 e TG pré-intervenção=168, IQR=151-200; pós-intervenção=137, IQR=94-177, p=<0.001 sofreram modificações significativas seis meses após a intervenção. Antes do estudo, nenhum dos pacientes apresentava níveis séricos normais para o CT, e apenas 12 pacientes (22,7%) tinham níveis séricos ótimo ou quase ótimos para o LDL. Seis meses após o início da intervenção, 45 pacientes (84,9%) alcançaram níveis séricos normais de CT e 50 (94,4%) níveis séricos ótimos ou quase ótimos de LDL. Os níveis séricos de HDL e as medidas antropométricas não sofreram modificações significativas.

**Conclusões:**

Aconselhamento dietético com prescrição de dieta individualizada baseada no cálculo do metabolismo basal mostrou-se efetivo no manejo da dislipidemia em pacientes transplantados hepáticos em seguimento ambulatorial. Assim, todos os pacientes transplantados hepáticos com dislipidemia devem ser incluídos em um programa de intervenção dietética sob orientação de nutricionista.

## INTRODUCTION

Liver transplantation (LT) is the standard of care in the treatment of patients suffering from acute and chronic end-stage liver disease and selected primary and metastatic cancer to the liver^14^. It is the second most performed type of organ transplant, surpassed only by kidney transplantation^6^. A significant improvement in quality of life and survival is expected following it, and LT recipients may reach up to 80% 5-year survival ^7, 13, 28^.

As immediate results of LT have improved, death from cardiovascular causes has acquired a prominent role, for which metabolic syndrome represents an important risk factor^21^. It consists of several metabolic disorders, including visceral obesity, diabetes mellitus, dyslipidemia and hypertension^12^. Up to a third of patients undergoing TH become obese in the period of three years after surgery^22^. Besides that, differently from cirrhotic patients who commonly present with low serum lipid levels, a high percentage of liver transplants have abnormally elevated serum lipid^4^. Dyslipidemia, one of the components of metabolic syndrome, is present in up to 70% of all LT recipients^17^, and constitutes an important risk factor for post-transplant cardiovascular morbidity and mortality^29^. 

Dietary management represents an area with a great research potential^16^ ^,^ ^25^. The potential benefits of dietary intervention would include prevention of cardiovascular death and increase satisfaction and quality of life of liver transplant patients^15, 25^. No prior studies have demonstrated any dietary intervention that could effectively control dyslipidemia in these patients. 

The aim of this study was to verify the effects of a dietary intervention comprising dietary counseling with prescription of an individualized diet on the lipid profile of post-liver transplantation patients with dyslipidemia.

## METHODS

This research was approved by Institutional Research Board from Hospital de Clínicas de Porto Alegre and carried out according to the ethical guidelines outlined by The Transplantation Society and have involved no commercial transactions or other unethical practices in obtaining donor organs. 

### Population

All liver transplant patients using whole liver graft from a deceased donor at our Institution between 2002 and July, 2014 and followed for dyslipidemia were enrolled. Inclusion criteria were being at least two months after LT, age >18, and suffering from dyslipidemia without the use of lipid-lowering drugs. Pregnant patients, drug users, and patients suffering from any physical or neurological disability that prevented them from undergoing nutritional or anthropometric evaluation or obtaining the informed consent were not included. Dietary consultations and data collection were performed at the Clinical Research Center of the Federal University of Rio Grande do Sul from March, 2014 to May, 2015 by the first author of this study (Pinto, AS).

### Logistics

Patients were followed for the period of six months, in three meetings (baseline-, 3^rd^- and 6^th^ -month meetings). Identification, clinical, anthropometric and biochemical data were recorded. Besides, the self-report about the practice of physical exercises were also included in each patient's record. Serum levels of total cholesterol (TC), HDL-cholesterol (HDL), LDL-cholesterol (LDL) and triglycerides (TG) were measured. Biochemical exams were analyzed at baseline (pre-intervention) and in the last meeting (post-intervention).

 Anthropometric evaluation, food history with the use of a 24 h recall, body composition analysis through bioimpedance and resting energetic expenditure though indirect calorimetry were recorded at baseline. After the settlement of resting energy expenditure, the total calorie value of the diet was calculated for each patient. 

Diet review, anthropometric evaluation and 24 h recall were performed on 3^rd^ and 6^th^ month meetings. Bioimpedance and indirect calorimetry also were repeated on 6^th^ month meeting. 

### Anthropometric evaluation

It was performed by a single researcher and included: weight, height, body mass index, circumference waist, neck circumference, triceps skinfold and subscapular skinfold. According to body mass index in kg/m² for adults were classified as: low weight <18,5; eutrophic: 18,5-24,9; overweight: ≥25; pre-obese: 25,0 to 29,9; obese I: 30,0 to 34,9; obese II: 35,0 to 39,9; and obese III: ≥ 40,0. Elderly (aged <65 years-old) were classified as following: low weight: ≤22; eutrophic: >22 and < 27; and overweight: ≥27^30^.

Circumferences were measured with a non-flexible tape-measure. The circumference waist was measured at the midpoint between the lower costal margin and the iliac crest. It was established as cutting point for increased cardiovascular risk circumference waist, equal or above 94 cm for men and 80 cm for women. Men with a circumference waist, above 102 cm and women above 88 cm were classified as possessing a very increased cardiovascular risk^9^. Neck circumference was measured with the patient facing the evaluator and the tape positioned in the middle of the neck between the mean cervical spine and the anterior neck (for men possessing a laryngeal prominence, measurements were performed an inch below laryngeal prominence). The neck circumference cut off levels for classifying individuals as overweight were 37 cm for men and 34 cm for women. For obesity, neck circumference measures were 39.5 cm or higher for men and 36.5 cm or higher for women^3^. 

For measuring skinfolds, a Cescorf(r) adipometer was utilized. Measurements were performed on the right side of the body, with the subject resting at orthostatic position. For the triceps skinfold, the anatomical reference was the midpoint between the lateral projection of the acromion process of the scapula and the lower edge of the olecranon. The fold was vertically pinched to the longitudinal axis, in the rear arm^24^. Regarding the subscapular skinfold, the anatomical reference was 2 cm below the lower angle of the scapula. The adipometer was placed in the natural fold direction, obliquely downward and laterally to the longitudinal axis of the body at a 45° angle^24^.

### Food history

Food history was performed through of 24 h recall, aiming to quantify all the food ingested at home in a regular day. It was assessed before baseline meeting and also before the two subsequent meetings (at 3^rd^ and 6^th^ follow-up months) ^11^. 

### Evaluation of body composition

#### Bioimpedance 

The patients were told to fast for 8 h before the procedure and to abstain from performing any physical activity and from alcohol and caffeine on the 24 h preceding the exam. Patients were also told to avoid heavy meals for at least 4 h before the test. They should not wear watches, armbands, earrings or necklaces during the test. A body composition analyzer, model Bodystat(r) 1500, was utilized. This method employed four little electrodes attached to their right hand and fist, and to the right ankle and foot. As standard, the bioimpedance measures were performed on the right side of the body^19^. The device was connected to the two pairs of electrodes and a low-voltage current was passed through the body, measuring the electrical resistance and reactance^19^. Similarly, the lean body mass, fat body mass, basal metabolic rate and total body water measures were obtained.

#### Indirect calorimetry (IC) 

The indirect calorimetry is a non-invasive method which determines the energy needs and the rate of substrate utilization from the volume of oxygen consumption and the carbon dioxide production, obtained through analysis of air inhaled and exhaled by the lungs^2^. In patients with liver diseases, it is suggested that other factors may be involved in the basal metabolism alterations as a result of the chronic liver disease or the provided immunosuppressive treatment^10^. Assessment of the resting energy metabolism relative to a standard is performed based on the basal metabolism comparison measured by the standard method with the predictive formulas^13^. The basal metabolic was measured in a thermoneutral environment through indirect calorimetry (Metabolic Gas Analyzer VO 2000, Software Aerograph Breeze, Medical Graphics - Cardiorespiratory Diagnostic Systems), after a fasting period of at least 8 h. The system was calibrated according to before each measurement. The oxygen consumption and the carbon dioxide production were measured with a patient in supine position during 25 min (including the initial time of 5 min).

### Biochemistry evaluation

Blood lipid profile included serum levels of TC, HDL, LDL and TG, being measured at baseline (pre-intervention) and in the last meeting (post-intervention).

### Dietary intervention

Dietary counseling was individualized considering the needs of each patient. The dietary regimen consisted of detailed description of the food replacements, times and daily amounts in household measures. Calorie needs were based on the resting energy expenditure obtained by the standard method for each patient plus +35 kcal/kg/day for early LT recipients and +30 kcal/kg/day for late LT recipients^23^. The individualized diet was prescribed following recommendations of specific guidelines for prevention of atherosclerosis, and consisted of 25% of the energy value in total fat, ≤7% of the total energy value in saturated fat and <200 mg/day of cholesterol^27^. 

The diet and the dietary counseling were considered effective in those patients who reached a TC reduction to less than 200 mg/dl, TG less than 150 mg/dl, LDL less than 130 mg/dl and HDL increase to serum levels higher than 60 mg/dl.

### Statistical analysis 

Association of dietary intervention to each TC, LDL, HDL and TG was evaluated through univariate analysis. Qualitative variables were compared using chi-square test. Quantitative variables were submitted to a normality test (Shapiro-Wilk). Quantitative variables with normal distribution were presented as mean and standard deviation, and compared through paired t-test. Quantitative variables with asymmetric distribution were presented in median and quartiles, and compared through Wilcoxon Signed-rank Test. p<0.05 was considered as statistically significant. 

Potential confounding factors to the effect of diet on TC, LDL, HDL and TG were analyzed through univariate analyses. All potential confounding factors associated with improvement in any of the lipid parameters (p<0.1) were evaluated together with the effect of dietary intervention on TC, LDL, HDL and TG through the model Estimations Generalized Equations. In this analysis, the covariance matrix with robust estimator was used, an unstructured working correlation matrix and a normal distribution or gamma with identity or logarithmic link function when needed. Confidence intervals (95% CI) were calculated as needed. Data were analyzed using SPSS - version 18 (IBM Company, USA).

## RESULTS

Fifty-three of all 56 patients concluded the study (two patients were lost to follow-up and the remaining patient died during the study period). Mean age was 59±10.10 years. There were 29 men (51.8%) and 27 women (48.2%). Mean time after LT was 46.5 months. The most common associated conditions were hypertension (n=29, 51.8%) and type 2 diabetes (n=25, 44.6%); 25 (44.6%) were obese; 35 (62.5%) had sedentary lifestyle; 24 (42.9%) were on steroid therapy; 17 (30.4%) were on diuretic therapy; 8 (14.3%) were on beta-blockers use; 47 (83.9%) were on tacrolimus treatment; 37 (66.1%) were on mycophenolate use; and 33 (58.9%) were on antihypertensive drug therapy (Table 1).

**TABLE 1:**
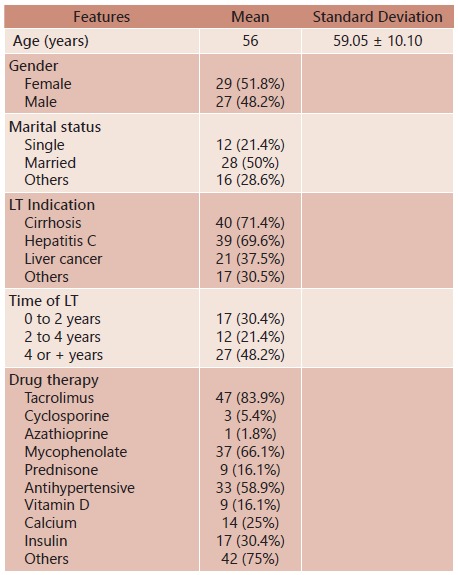
General features of the patients post-liver transplantation

LT=liver transplantation

At baseline, 38 patients (67.9%) did not practice any physical activity, and 18 patients (32.2 %) exercised once or twice a week. At the end of the follow-up six months, 25 (47.2%) kept on a sedentary lifestyle and 28 (52.8%) performed some degree of physical activity. 

**FIGURE 1:**
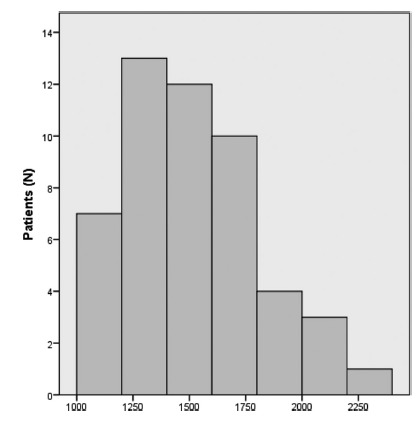
Pre-intervention resting energy expenditure: distribution as calculated by indirect calorimetry

Mean total pre-intervention resting energy expenditure was 1533.16±290.7 kcal. The mean post-intervention resting energy expenditure was 1504±275.5 kcal (p=0.142). The distribution of the resting energy expenditure of each patient is displayed in Figure 1. Mean total energy value of the prescribed diet based on the patients' resting energy expenditure was 1853.57±218.2 kcal.

At the end of the six follow-up months, there was a statistically significant reduction to levels considered clinically normal for mean TC levels, mean LDL levels and median TG levels for the study population (Table 2). Pre-intervention means TC was 238.9±30, and post-intervention mean TC was 165.1±35 (p<0.001). Pre-intervention mean LDL was 154±33, and post-intervention mean LDL was 90±29 (p<0.001). Pre-intervention median TG was 168 (IQR=51-200), and post-intervention median TG was 137 was (IQR=94-177, p=<0.001). HDL was not modified (Table 2). 

**TABLE 2:** Pre- and post-diet lipid profile

Parameter	Before	After	p value
TC¹	238.83 ± 30.14	165.07 ± 35	< 0.001
LDL¹	153.80 ± 33.08	89.91 ± 28.94	< 0.001
HDL²	44.50 [31.8; 55.8]	41.0 [33.0; 56.5]	= 0.302
TG²	167.5 [151.2; 200]	137.0 [94.0; 177]	< 0.001

Data with normal distribution are expressed in mean ± standard deviation and data with asymmetric distribution are expressed as median (interquartile range). 1-Paired t-test; 2- Wilcoxon test; TC=total cholesterol; LDL=low-density lipoprotein; HDL=high-density lipoprotein; TG=triglycerides.

At baseline, none of the patients had desirable serum TC levels (Table 4). After intervention, 45 out of all 53 patients (84.9%) had normal TC. Before intervention, only 12 patients (22.7%) had optimal or near optimal LDL levels. After intervention, 50 patients (94.4%) had optimal or near optimal serum LDL. Additionally, there was a four-time increase in the amount of patients presenting with normal levels following intervention (Table 3).

**TABLE 3:**
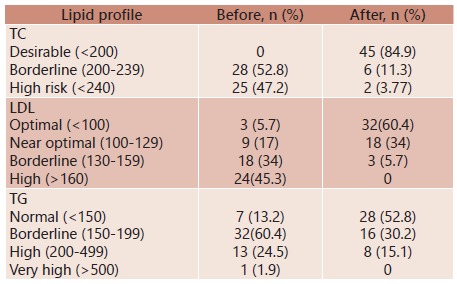
Distribution of pre- and post-diet lipid profile according (stratified by serum levels)

**TABLE 4:**
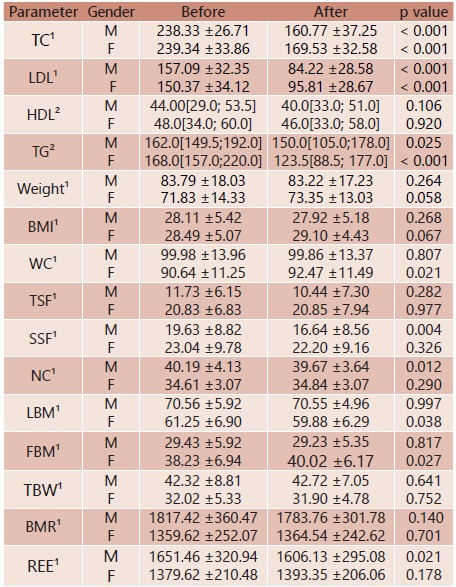
Lipid profile and nutritional status stratified by gender

Data with normal distribution are expressed in mean ± standard deviation and data with asymmetric distribution are expressed as median (interquartile range). 1- Paired t-test; 2- Wilcoxon test; F=female; M=male; TC=total cholesterol; LDL=low-density lipoprotein; HDL=high-density lipoprotein; TG=triglycerides; BMI=body mass index; WC=waist circumference; TSF=triceps skinfold; SSF=subscapular skinfold; NC=neck circumference; LBM=lean body mass; FBM=fat body mass; TBW=total body water; BMR=basal metabolic rate; REE=resting energy expenditure.=

Stratifying per gender, similar results were obtained (Table 4). Both men and women were classified as overweight (body mass index ≥25 kg/m²) pre- and post-intervention (Table 4). For the circumference waist both pre- and post-intervention, men were considered as being at an increased cardiovascular risk and the women in very increased cardiovascular risk. Basal metabolic rate and resting energy expenditure were higher in the men than in the women (Table 4). Except for the subscapular skinfold, anthropometric measures (weight, body mass index, waist circumference, neck circumference, triceps skinfold, subscapular skinfold and body composition (lean body mass, fatty body mass, total body weight and basal metabolic rate did not show significant changes following intervention (Table 5). 

**TABLE 5:** Pre- and post-diet nutritional status of the patients

	Before, Mean ± Std. Dev	After Mean ±Std. Dev	p value
Weight (kg)	78.05 ±17.28	78.48 ±16.00	0.357
BMI (kg/m²)	28.29 ± 5.21	28.49 ±4.82	0.286
WC (cm)	95.50 ±13.45	96.32 ±12.93	0.072
TSF (mm)	16.10 ±7.89	15.44 ±9.19	0.348
SSF (mm)	21.27 ±9.36	19.31 ±9.20	0.004
NC (cm)	37.50 ±4.59	37.35 ±4.14	0.303
LBM (%)	66.43 ±7.73	65.44 ±7.75	0.236
FBM (%)	33.35 ±7.60	34.41 ±7.88	0.209
TBW (L)	37.61 ±8.72	37.53 ±8.12	0.753
BMR (kcal)	1609.39 ±377.53	1582.54 ±344.763	0.255
REE(kcal)	1533.16 ±290.7	1504.0 ±275.48	0.142

Paired t-test. BMI=body mass index; WC=waist circunference; TSF=triceps skinfold; SSF=subscapular skinfold; NC=neck circumference; LBM=lean body mass; FBM=fat body mass; TBW=total body water; BMR=basal metabolic rate; REE=resting energy expenditure

The effect of dietary intervention on TC levels was not influenced by any of the following factors: use of tacrolimus, use of mycophenolate, use of prednisone, use of any anti-hypertensive drug, vitamin D therapy, calcium therapy, diabetes on insulin therapy, obesity, sedentary life style, diabetes, and diuretic therapy, and practice of physical exercise. The effect of the diet on TG levels was influenced by use of insulin (p<0.001) and use of diuretics (p=0.003), and also by use of antihypertensive medication (p=0.026). The effect of the dietary intervention on LDL levels received influence from the use of prednisone (p=0.015). The time frame after transplantation (0-2 years, 2-4 years, 4 or + years) did not influence the effect of the diet intervention on lipid profile (TC, TG, LDL). 

## DISCUSSION

This study evaluated the results of a dietary intervention consisting of dietary counseling with prescription of individualized diet to a cohort of 53 liver transplant patients suffering from dyslipidemia. The diet was based on accurate resting energy expenditure values estimated by indirect calorimetry, the gold standard method for basal metabolism measurements. This intervention yielded significant improvements on lipid profile (TC, LDL and TG) for this cohort. The improvements on TC, LDL and TG were statistically significant, "normalizing" mean TC, mean LDL and median TG for this cohort. At baseline, none of the patients had desirable TC levels. After intervention, 45 out of all 53 patients (84.9%) had normal TC. Before intervention, only 12 patients (22.7%) had optimal or near optimal LDL levels. After intervention, 50 patients (94.4%) had optimal or near optimal serum LDL. Additionally, there was a four-time increase in the amount of patients presenting with normal levels following intervention. HDL profile was not improved by dietary intervention. 

A recent study evaluated 23 LT recipients (16 females and 7 males) with BMI >27 kg/m² and suffering from dyslipidemia. Patients were evaluated before and after six months of dietary intervention based on a diet recommended by the American Heart Association. Following dietary intervention, TC levels were reduced (p<0.05). However, when the data was stratified by gender, both TC and LDL were reduced only for male patients. There were no significant reductions on TG and HDL for this population^20^. Another study evaluated the effect of a 12-month diet with total fat below 30% and cholesterol below 300 mg/day on the lipid profile of 46 kidney transplant recipients. A significant weight loss and BMI reduction along with concurrent improvements in TC levels were obtained. No significant change on TG was obtained^14^. 

Physical activity levels of liver transplant patients usually are significantly inferior to those from general population^7^. A study evaluating 136 patients after liver transplantation verified that most patients did not go back to work and/or remained on a sedentary life style after this procedure^26^. The current study showed that there was an increase in practice of physical activities from 32.2% to 50 % during study period. Similarly, another study evaluating liver transplant patients showed that 51% of the patients reported to engage in some physical activity, with more than 27% of the population performing more than 150 minutes per week^16^. It is know that the exercise capacity is limited before and after LT, suggesting that combining dietary intervention and physical activity protocols would help improving both anthropometric parameters and lipid profile. 

Immunosuppressive drugs have been associated to post-transplant metabolic syndrome and to an increased cardiovascular risk for liver transplant patients^10^ ^,^ ^16^. Death from cardiovascular cause has acquired a prominent role in LT recipient population, for which metabolic syndrome also represents an important risk factor^21^. Although yielding significant improvement on lipid profile, the dietary intervention evaluated in the present study was not effective in improving anthropometric parameters, with most patients remaining overweight after the end of this study. Other studies also show similar results^1^ ^,^ ^18^. It remains unclear whether the physical exercise levels were not enough for improving anthropometric parameters or the observation period was too short for obtaining significant weight loss. 

This improvement in lipid profile may have happened secondarily to dietary counseling along with the diet. Besides, the fact that the dietary counseling has been individualized and resting energy expenditure was measured by indirect calorimetry reinforces the importance of having a diet prescribed in an individualized and accurate fashion.

TC levels did not suffer any effect of potential confounders. In other words, TC reduction levels was influenced only by the dietary intervention. For TG levels, diabetes on insulin therapy was a confounding factor, potentially influencing the effect of the dietary intervention on serum TG levels. Similar findings were reported elsewhere^1^. Use of diuretics also may have influenced the effect of the diet on improvements of TG levels. However, prior studies reported LT recipients having their lipid profile worsened by the use of diuretics and beta blockers. In addition to promote an increase in TG, diuretics may also cause glucose intolerance, raising the risk for diabetes^5^. The effect of this dietary intervention on LDL might have been influenced by use of prednisone. No prior study had reported similar results. Instead, the studies show that prednisone is an immunosuppressive drug which promotes metabolic disorders, mainly dyslipidemia, increasing the lipid levels and not reducing them^8^. 

As a limitation to the present study stands out the absence of a control group. Although improvements on TC, LDL and TG could have occurred secondary to other factors other than dietary intervention, the main confounding factors were analyzed by the Generalized Estimating Equations model. Thus, it seems that the majority of the improvements on lipid profile can be attributed to the dietary intervention. Another limitation of this study is its short follow-up period. However, considering the improvement to the lipid profile occurred in a short time span, it is likely that a longer study period would reflect on further important improvements, including anthropometric ones.

## CONCLUSION

Dietary counseling with prescription of individualized diet based on calculated resting energy expenditure by indirect calorimetry was able to manage dyslipidemia in most LT recipients evaluated in this study. We suggest all dyslipidemic LT recipients be enrolled on a dietary program. 
